# A novel application of Raman spectroscopy to monitor cell wall removal during protoplasting in grapevine

**DOI:** 10.3389/fpls.2026.1940456

**Published:** 2026-09-18

**Authors:** Edoardo Bertini, Erica D’Incà, Haruna Chikaguchi, Marco Giarola, Stefania Zattoni, Sara Lissandrini, Alessandra Amato, Mario Pezzotti, Giuseppe Pezzotti, Sara Zenoni

**Affiliations:** 1 EdiVite S.r.l., San Pietro Viminario, Padua, Italy; 2 Ceramic Physics Laboratory, Kyoto Institute of Technology, Kyoto, Japan; 3 Department of Computer Science, University of Verona, Verona, Italy; 4 Department of Biotechnology, University of Verona, Verona, Italy; 5 Biomedical Engineering Center, Kansai Medical University, Osaka, Japan

**Keywords:** cell wall degradation, cellulose bands, grapevine, plant regeneration, protoplasts, Raman spectroscopy, transfection

## Abstract

**Introduction:**

Grapevine is one of the most widely cultivated and economically important perennial fruit crops worldwide. However, its genetic improvement is severely constrained by a long juvenile phase and a high degree of recalcitrance in *in vitro* regeneration and biotechnology applications. Recently, the feasibility of DNA-free genome editing through a protoplast-based platform has been demonstrated in grapevine. However, the successful application of this approach requires the optimization of complex and genotype-dependent protocols, ranging from tissue culture establishment and protoplast isolation to cell regeneration and whole-plant recovery.

**Methods:**

Here, we investigated Raman spectroscopy (RS) as a noninvasive and label-free approach to monitor cell wall degradation during protoplast isolation in two grapevine cultivars, *Vitis vinifera* cv. Syrah and cv. Thompson Seedless. The RS-based assessment was integrated into a protoplast-to-plant workflow, which was subsequently evaluated through protoplast cultivation and whole-plant regeneration.

**Results:**

For both cultivars, RS revealed specific cellulose-, xyloglucan-, and lignin-related spectral features in embryogenic calli that were absent or reduced in isolated protoplasts following enzymatic digestion. These spectral changes were supported by Fluorescent Brightener 28 staining and successful polyethylene glycol PEG-mediated GFP transfection, confirming that cell wall degradation was functionally sufficient for exogenous material delivery and downstream applications. The isolated protoplasts retained developmental competence, as demonstrated by somatic embryogenesis and whole-plant regeneration in both cultivars.

**Discussion:**

These findings indicate that RS provides a standardized, label-free quality-control step for monitoring the protoplasting process in grapevine without compromising cell integrity. By providing detailed molecular profiling before and after enzymatic digestion, RS allows objective validation of cell wall removal. Successful plant regeneration serves as a biological downstream validation, confirming that the isolated protoplasts, in which cell wall digestion was demonstrated by RS, preserved developmental competence and totipotency. Integrated into protoplast workflows, RS enhances reproducibility and efficiency for downstream biotechnological applications, including DNA-free genome editing in recalcitrant woody species.

## 1 Introduction

Grapevine (*Vitis vinifera* L.) is one of the most important fruit crops as it is widely cultivated, and the winemaking industry has a substantial economic importance worldwide (Martínez-Esteso et al., 2013; Martínez-Lüscher et al., 2025). The quality of grape berries and their subsequent products, foremost wine, is strongly associated with a delicate balance of their metabolite levels, including polyphenols, organic and amino acids, volatiles and flavor precursors, and polysaccharide contents. Color, aroma, flavor, and other in-mouth sensations of wine are characterized by these components, which are influenced by various pre- and postharvesting conditions, including biotic and abiotic stress (Kuhn et al., 2014). Crop improvement in *V. vinifera* has always been of interest, aimed at controlling the adverse effects of biotic and abiotic stress and promoting desirable characteristics, and inducing disease resistance (Armijo et al., 2016; Paineau et al., 2025).

Genetic transformation, as the main tool to facilitate the transfer of desirable traits, still has some technical issues, and it is not efficient in grapevine (Liang et al., 2026). However, examples of genetic modification of grapevine have been reported in several studies mainly to improve biotic and abiotic resistance (Vidal et al., 2006; Dai et al., 2016; Cui et al., 2023; Liang et al., 2026). Despite these achievements, the lack of social acceptance and strict and costly regulations for genetically modified crops have strongly limited the widespread cultivation of transgenic grapevines in many countries (Sprink et al., 2016). The emerging legislative frameworks surrounding New Genomic Techniques (NGTs), specifically the NGT-1 category, are opening new horizons by treating transgene-free edited plants equivalently to conventionally bred varieties, thus significantly reducing commercialization hurdles (https://eur-lex.europa.eu/eli/reg/2026/1388/oj).

The protoplast platform has become a valuable system for physiological, biochemical, and molecular research, especially for genetic transformation of plants, including the DNA-free genome editing application for the production of NGT-1 plants (Reyna-Llorens et al., 2023). Protoplasts are naked plant cells from which all or part of the cell wall has been removed by mechanical or enzymatic treatments, providing a unique single-cell system (Davey et al., 2005). The absence of the cell wall facilitates gene transfer into the plant genome and avoids the production of chimeras. However, one of the major limitations of using protoplasts in plant breeding programs in recalcitrant plants, such as grapevine, is limited totipotency, which reduces the rate of plant regeneration. Optimization of the protocols for culture initiation and maintenance, protoplast isolation, and plant regeneration is indispensable to exploit protoplasts for effective transformation and genetic improvement of grapevine. In the last three decades, many efforts have been made, and protoplast-to-plant regeneration has been successfully described in some grapevine cultivars, such as Seyval Blanc (Reustle et al., 1995), Koshusanjaku (Zhu et al., 1997), and Garganega and Sangiovese (Bertini et al., 2019).

Recently, grapevine protoplasts from several cultivars have been successfully used for CRISPR/Cas9 genome editing through PEG-mediated transfection or lipofection. These approaches can be implemented using standard laboratory equipment and may enable the generation of DNA-free edited grapevine plants potentially qualifying as NGT-1 (Scintilla et al., 2022; Najafi et al., 2023; Gambino et al., 2024; Tricoli and Debernardi, 2024; Bertini et al., 2025; Böttcher et al., 2025).

The protoplast-to-plant system and the application of DNA-free genome editing have opened new horizons in plant genetic improvement (Hsieh et al., 2026), but one of the crucial steps in this process that has received little attention is cell wall digestion and accurate resynthesis, which can boost efficiency in both transformation and plant regeneration. Polysaccharides, including cellulose, hemicellulose, and pectin, are fundamental cell wall components in vascular plants, while structural proteins make up about one-tenth of the cell wall components (Malacarne et al., 2024). Moreover, cell walls are modified by wall-bound compounds that confer resistance to enzymatic degradation, such as P-coumaric acid and ferulic acid, two compounds known to play a role in cell wall resistance and are abundant in grape species (Zhang et al., 2021). Specific molecular analyses are fundamental to investigating the plant cell wall structure and composition and to verify polymer degradation during protoplast isolation.

In recent years, Raman spectroscopy (RS) has been demonstrated to be a powerful analytical tool in biology for both chemical analysis and imaging. It has been successfully applied to whole human tissues, such as skin (Miyamori et al., 2021), bone (Pezzotti et al., 2017), and teeth (Marin et al., 2020), as well as to human cells (Puppulin et al., 2017; Pezzotti et al., 2018) and human pathogens (Pezzotti et al., 2021; Boschetto et al., 2018). Furthermore, Raman spectroscopy has been extensively used in plant biology to study plant cell wall architecture and components (Agarwal, 2006; Schmidt et al., 2010; Zeise et al., 2018) and to perform *in vivo* metabolic profiling across different plant tissues (Qin et al., 2011; Mandrile et al., 2022). Micro-Raman spectroscopy, in particular, uses a light microscope and piezomotors to achieve higher spatial resolution (up to 1 μm) and resolve cell membranes and organelles (Han et al., 2010; Szymańska-Chargot et al., 2016).

In the present study, we investigated whether RS could be used as a non-invasive, label-free tool to monitor cell wall degradation during grapevine protoplast isolation. To our knowledge, this is the first study to apply RS as a spectral quality-control method for protoplast preparation in grapevine. To evaluate the suitability of RS-based assessment within a functional protoplast workflow, the approach was applied to two economically relevant grapevine cultivars, cv. Syrah and cv. Thompson Seedless, followed by protoplast cultivation and whole-plant regeneration. The resulting protoplast-to-plant system therefore served as a downstream validation of protoplast efficiency and developmental competence.

## 2 Materials and methods

### 2.1 Plant material and induction of embryogenic cultures

Embryogenic cultures were initiated following the experimental protocols described in Bertini et al. (2025). Embryogenic calli of cv. Thomson Seedless were induced from young unopened leaves of *in vitro* shoot tip cultures. Leaves were placed with the abaxial side up on NB2 medium (**Supplementary Table 1**) and incubated in the dark at 28°C for 3–4 months. Embryogenic calli of cv. Syrah were initiated from inflorescences derived from fruiting cuttings. Inflorescences were sterilized by immersing them in 40 mL of 3% sodium hypochlorite containing 0.01% Tween 20 for 10 min with continuous shaking, followed by three 5-min washes in sterile distilled water. Stamens (anthers with intact filaments) were carefully separated from the cap and pistil before being placed on PIV medium (**Supplementary Table 1**). After 2–3 months, embryogenic cultures of both cultivars were transferred to C1^P^ medium (**Supplementary Table 1**) and subcultured in the same medium every 4 weeks.

### 2.2 Protoplast isolation

Protoplasts were isolated from embryogenic calli as described by Bertini et al. (2025). In summary, for protoplast isolation from embryogenic calli, after a subculture to C1^P^ medium 7–10 days prior to isolation, the calli were incubated in the digestion solution (**Supplementary Table 2**) for 6 h and subsequently purified by filtration and washing (**Supplementary Table 2**). The efficiency of cell wall digestion was checked by staining protoplasts with 2 μM Fluorescent Brightener 28 (Santa Cruz Biotechnology, Dallas, TX, USA) under UV light, and protoplast viability was determined by staining with 0.1 mg/mL fluorescein diacetate (FDA; Sigma-Aldrich) under UV light. Protoplasts were visualized using two different microscopes: a Leica TCS SP5 AOBS confocal microscope (Argon laser, 514 nm excitation source, 550–570 nm collection bandwidth, auto gain) and a DM2500 microscope (Leica Microsystems, Wetzlar, Germany) equipped with UV lamp and filter sets composed of an excitation filter (470/40 nm) and a barrier filter (525/50 nm) for FDA and an excitation filter (340–380 nm) and a barrier filter (LP 425) for Fluorescent Brightener 28.

### 2.3 Statistical analysis

All experiments were performed using three independent biological replicates (*n* = 3), as reported in **Supplementary Figure 1**, and data are presented as mean ± standard deviation (SD). Differences between cultivars at the same protoplast concentration were assessed using an independent-samples Student’s *t*-test. Statistical significance was set at ^*^p < 0.05, ^**^p < 0.01, and ^***^p < 0.001, while *p* ≥ 0.05 was considered not significant (ns). Differences among protoplast concentrations within each cultivar were assessed using a one-way ANOVA test (*α* = 0.05) followed by a Tukey’s HSD *post hoc* test.

### 2.4 Raman spectroscopy and data analysis

The Raman spectra of embryogenic callus and protoplasts were obtained using a DXR2 Raman Microscope (Thermo Fisher Scientific, Waltham, MA, USA) at room temperature. The 50× microscope objective (NA = 0.5) in backscattering geometry was used. The spectra were excited with a 785-nm IR laser diode and 15 mW of laser power at the sample. Raman scattering was collected with confocal illumination, using a grating of 830 lines/mm in the spectral range 200–1,800 cm^−1^ with an estimated resolution of 3.0–4.1 cm^−1^. The exposure time was 120 s, and the number of repetitions was eight for spectrum accumulation.

The measurements were acquired using a self-made sample holder. The sample holder consisted of an optically polished steel disc and a glass coverslip with a thickness of 150 µm, interconnected by a gasket. The hollow cylindrical gasket provides an airtight space, thus preventing dehydration of the sample. In the central part, a drop of sample was placed on the internal metallic holder. The thin glass layer of the coverslip allows Raman measurements without strong absorptions or spurious luminescence contributions. These effects were further reduced by confocal imaging, which allowed acquisition only from well-defined portions of the sample.

Each spectrum was frequency calibrated using the spectrum of silicon. All Raman spectra were analyzed using LabSpec 5.0 (HORIBA/Jobin-Yvon, Kyoto, Japan) and Origin 8.5 (OriginLab Co., Northampton, MA, USA) software.

### 2.5 Protoplast cultivation

Protoplasts were cultivated at three different concentrations (10^5^, 5 × 10^5^, and 1 × 10^6^ protoplasts/mL plating density) using the disc-culture method. Protoplasts were resuspended in P-SM (**Supplementary Table 1**), and four 1-mL drops were collected in plates for three replicates (12 drops in total). When the medium had solidified, Protoplasts-Liquid Medium P-LM (**Supplementary Table 1**) was added as a reservoir (**Supplementary Figure 1**). The P-LM was changed every 2 weeks with fresh medium. Cultured protoplasts were maintained in the dark at 28 °C. After 2–3 months of culture, well-shaped somatic embryos at the cotyledonary stage were formed and transferred to Somatic Embryos-Medium SE-M (**Supplementary Table 1**) and maintained in the dark for around 1 month to promote further development.

Protoplast regeneration steps were analyzed using an Olympus IX70 microscope.

### 2.6 Whole-plant regeneration

After around one month in SE-M, well-developed germinated embryos were transferred to the shooting medium. To identify the most efficient shoot-induction conditions, four types of media were evaluated by adding the synthetic plant hormone 6-benzylaminopurine (BAP) to the basal media Chée and Pool (C2D) Vitis medium C2D and MG1. The four conditions were C2D with no BAP, C2D plus 4 μM BAP (C2D + 4B), MG1 with no BAP, and MG1 plus 10 μM BAP (MG1 + 10B) (**Supplementary Table 1**). Germinated embryos were incubated for 5–6 weeks with a 16-h photoperiod, and regenerated shoots were transferred to Murashige and Skoog (MS) medium supplemented with 1-Naphthaleneacetic Acid (NAA) MSN or Rooting Induction Medium RIM (**Supplementary Table 1**) for root induction and whole-plant regeneration. Well-rooted plants were transferred to potting soil in the growth chamber under controlled conditions (27 °C/22 °C day/night temperature, 50% humidity, 16-h photoperiod) for acclimation. Acclimated healthy plants were finally transferred to the greenhouse.

### 2.7 PEG-mediated transfection

PEG-mediated transfection of protoplasts was performed as described by Bertini et al. (2025). Briefly, 200 μL of MMG solution (**Supplementary Table 2**) was used to resuspend protoplasts of both cultivars at a concentration of 10^5^ protoplasts/mL, and 50 μg of the pEGB3Ω2-35S::GFP::Tnos vector carrying the GFP marker gene (Sarrion-Perdigones et al., 2013) was added. An empty pDGB3Ω2 vector was used as a negative control. Subsequently, 210 μL of PEG solution (**Supplementary Table 2**) was added and immediately mixed with the protoplast suspension. After an incubation of 20 min at room temperature in the dark, protoplasts were washed by adding 950 μL of W5 solution (**Supplementary Table 2**) and centrifuged at 100×*g* for 3 min. Finally, 1 mL of W1 solution (**Supplementary Table 2**) was used to resuspend protoplasts, which were then incubated in the dark at room temperature overnight. The efficiency of transfection was evaluated at 24, 48, and 72 h posttransfection using an Olympus IX70 microscope equipped with a UV lamp and filter sets composed of an excitation filter (470/40 nm) and an emission filter (520 nm).

## 3 Results

### 3.1 Embryogenic calli induction

Embryogenic calli were initiated in *Vitis vinifera* cv. Thompson Seedless from unopened leaves of *in vitro*-grown plants (**Figures 1A, B**), while in cv. Syrah, they were initiated from stamens of fruit-cutting plants (**Figures 1D, E**). After around 10 weeks, explants produced two types of callus cultures, which were distinguished as cream-colored embryogenic callus and dark brown nonembryogenic callus (**Figures 1C, F**). Embryogenic calli were transferred to C1 medium for long-term maintenance.

**Figure 1:**
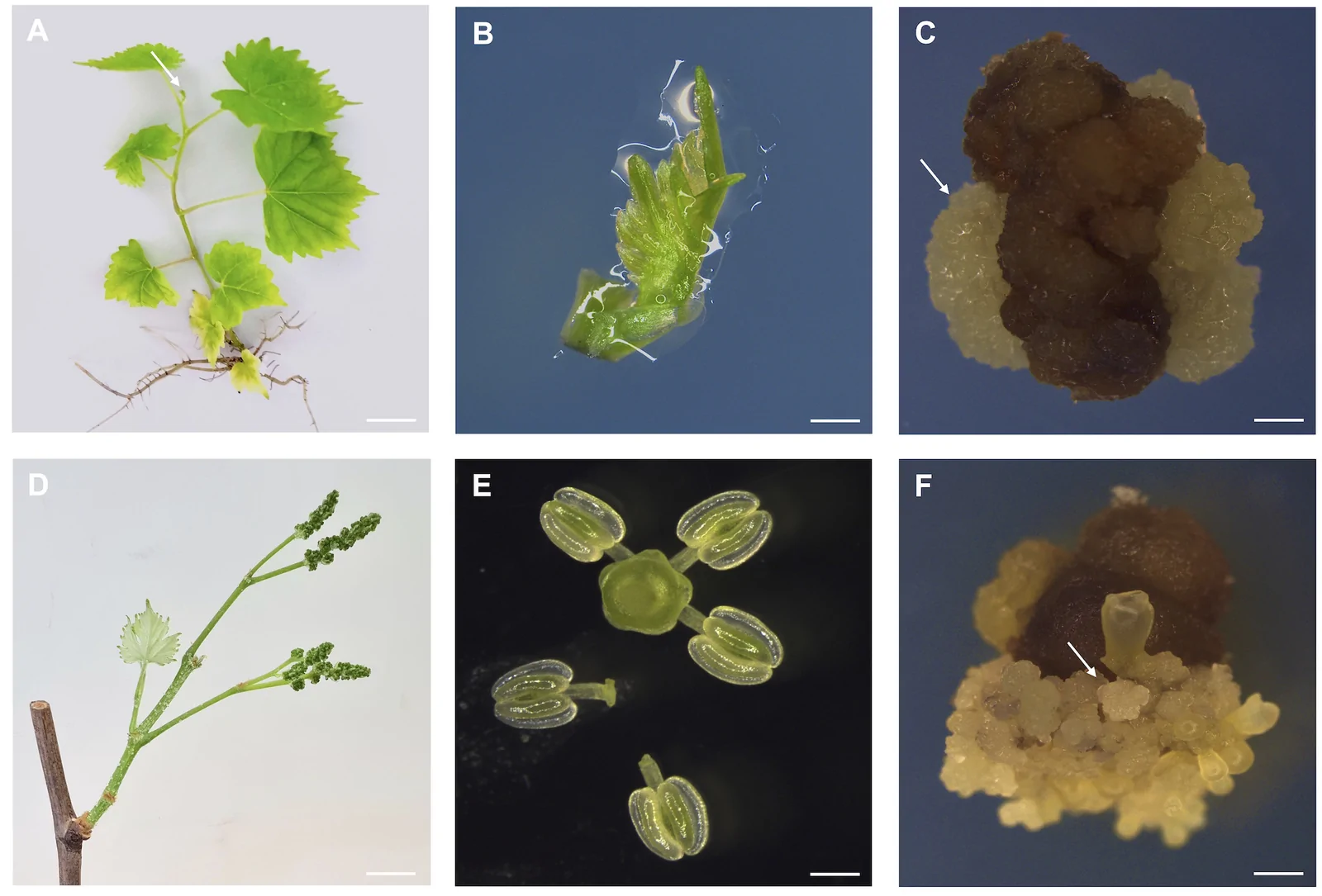
Embryogenic callus induction in *Vitis vinifera*. **(A)** *In vitro* plantlet of cv. Thompson Seedless. The arrow indicates the leaf used for embryogenic callus induction. Scale bar = 1 cm. **(B)** Unopened leaves of cv. Thompson Seedless. Scale bar = 0.1 cm. **(C)** Embryogenic calli (indicated by a white arrow) induced from unopened leaves of cv. Thompson Seedless in C1^P^ medium. **(D)** Inflorescences of fruit cuttings of cv. Syrah. Scale bar = 3 cm. **(E)** Mature stamens of cv. Syrah. Scale bar = 3 mm. **(F)** Embryogenic calli (indicated by a white arrow) induced from mature stamens of cv. Syrah in C1^P^ medium. Scale bar = 0.1 cm.

### 3.2 Protoplast isolation efficiency

Protoplasts were obtained from established and highly regenerative cultures of embryogenic calli of cv. Thompson Seedless and cv. Syrah after 7–10 days of subculture in C1^P^ medium (**Figure 2A**). Protoplast isolation was monitored by microscopy. Protoplasts started to appear 1.5 h after digestion, but many clusters of nondigested cells were still present. After 3 h, the number of protoplasts increased, even though many of them did not show the characteristic circular shape. Complete digestion was achieved 4.5 h after the start of digestion, and the resulting protoplasts were used for further analysis.

**Figure 2:**
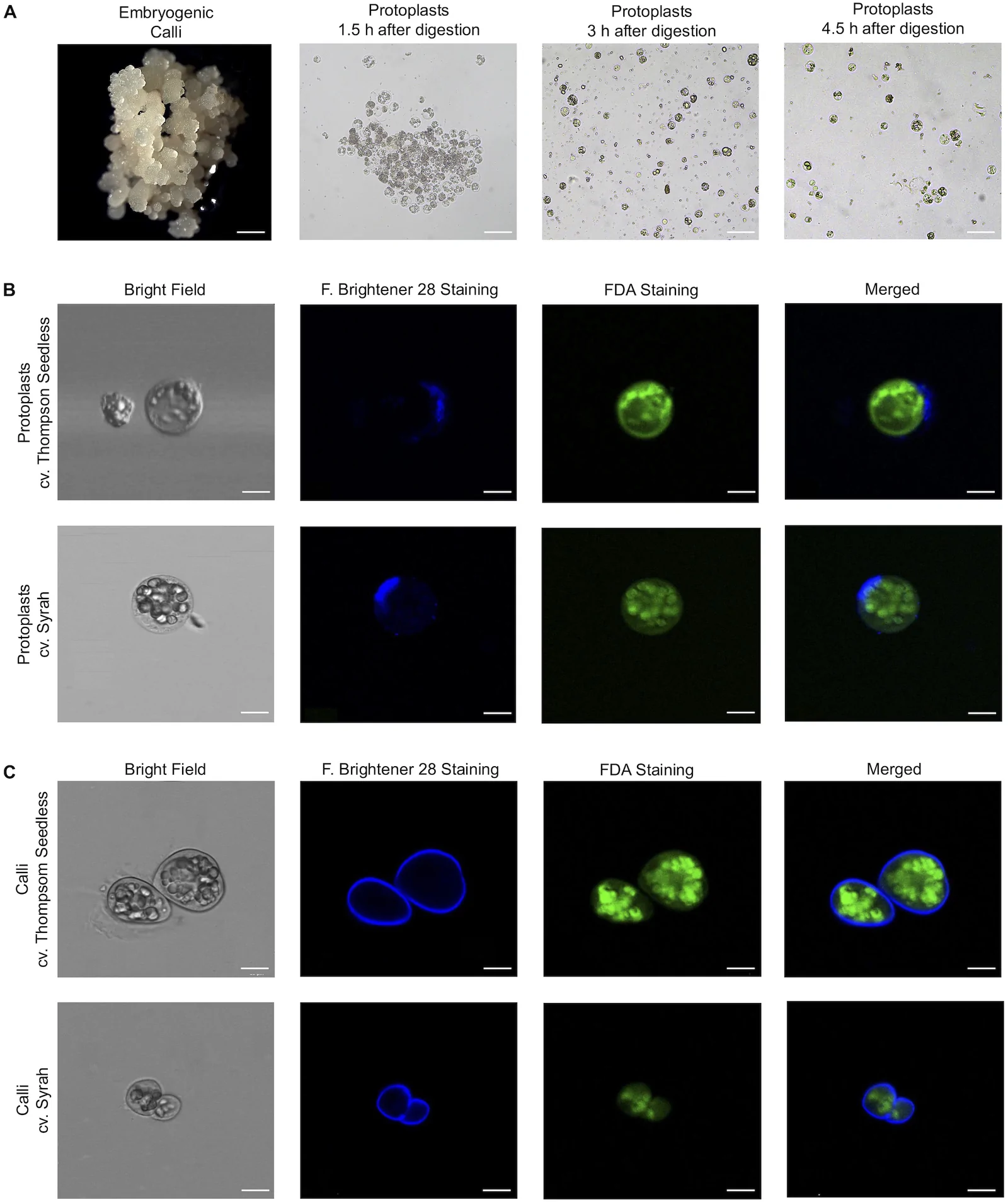
Protoplast isolation efficiency in *Vitis vinifera*. **(A)** Protoplast isolation from embryogenic calli. From the left: embryogenic calli (scale bar = 1 cm); isolated protoplasts at 1.30, 3, and 4.5 h after digestion. Scale bar = 20 µm. **(B)** Analysis of cell wall digestion efficiency and viability on isolated protoplasts of cv. Thompson Seedless and cv. Syrah using Fluorescent Brightener 28 and FDA staining methods, respectively. Scale bar = 5 µm. **(C)** Analysis of cell wall digestion efficiency and viability of calli of cv. Thompson Seedless and cv. Syrah using Fluorescent Brightener 28 and FDA staining methods, respectively. Scale bar = 5 µm.

The efficacy of plant cell wall digestion and the viability of isolated protoplast-derived cells of both cultivars were investigated by double Fluorescent Brightener 28 and FDA staining (**Figure 2B**; **Supplementary Figure 2**).

The cell wall was completely digested in protoplasts of both cultivars, as assessed by confocal microscopy, since no blue signal was detected. Only in a few cases were small fragments still visible.

Regarding the viability of isolated protoplasts, this feature reached an average of 85% in both genotypes. The negative control consisted of embryogenic callus cells treated with digestion solution without the enzymatic mixture and stained under the same conditions (**Figure 2C**). Confocal microscopy analysis showed a clear blue signal around the cells, indicating the presence of the plant cell wall.

The yield of protoplasts was 2.1 × 10^7^ and 6.9 × 10^7^ cells/g for cv. Thompson Seedless and cv. Syrah embryogenic calli, respectively.

### 3.3 Raman spectroscopy analysis

Embryogenic calli and isolated protoplasts of both genotypes were analyzed using Raman spectroscopy, an analytical technique that can measure molecular fingerprints based on the vibrational signatures of intracellular molecules in a nondestructive, label-free manner. Measurement spots were randomly selected, and 10 spectra were collected for embryogenic calli and protoplasts for each grapevine genotype.

**Figure 3** shows the average spectra of the embryogenic calluses and protoplasts for cv. Thompson Seedless (**Figure 3A**) and cv. Syrah (**Figure 3B**) in the 700–1,800-cm^−1^ spectral region. Band assignment has been summarized in **Table 1**, along with literature references. Overall, the spectra of calli and protoplasts share several spectral features, but clear differences in relative band intensity are observed, which are associated with the presence or absence of the cell walls.

**Figure 3:**
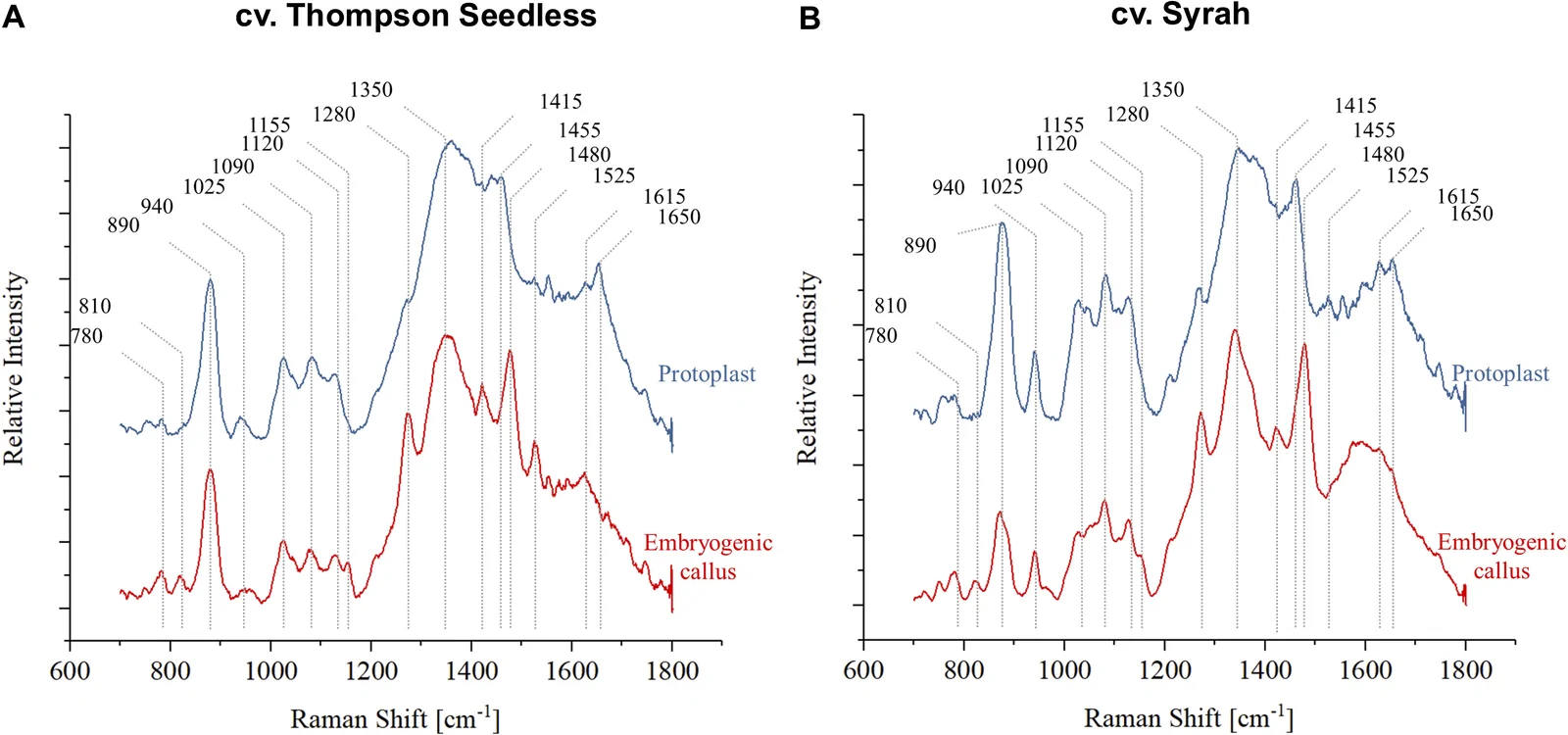
Raman spectra. **(A)** Spectra of embryogenic calli and protoplasts of cv. Thompson Seedless. **(B)** Spectra of embryogenic calli and protoplasts of cv. Syrah.

**Table 1:** Raman band positions (cm^−1^) and corresponding molecular assignments for the embryogenic calli and protoplast spectra presented in **Figure 3**.

Position	Assignment	Molecule	Reference
780	Pyranose ring deformation C−O−C skeletal bending	Xyloglucan	Szymańska-Chargot et al. (2016)
810	C−O−C bending in β-(1→4)-glycosidic bridge	Glucomannan	Volkov et al. (2019)
890	C1−O−C4 stretching of α-(1→4) glycosidic bond	Xyloglucan	Szymańska-Chargot et al. (2016)
		Pectin	Almeida et al. (2010)
940	C−O−C + C−O−H + C−O	Starch	Almeida et al. (2010)
1026	C−O stretching	Cellulose	Yu et al. (2007)
1090	Skeletal C−O−C stretching of the β-(1→4)-glycosidic linkage	Cellulose, hemicellulose	Zhang et al. (2017)
1129	Asymmetric C−O−C stretching	Cellulose	Zhang et al. (2026)
		Starch	Almeida et al. (2010)
1155	C−O−C stretching of the β-(1→4)-glycosidic bridge	Cellulose	Stevanic and Salmen (2009)
1272	Aryl−O phenolic ether stretching coupled with C−O stretching in methoxy groups	Lignin	Zhang et al. (2017)
1347	C−H in-plane bending	Starch	Almeida et al. (2010)
1415	CH_2_ scissoring of hydroxymethyl group	Cellulose	Stevanic and Salmen (2009)
1455	CH_2_ methylene scissoring and CH methine deformation	Pectin (low DE)	Szymańska-Chargot et al. (2016)
1480	CH_2_/CH bending	Xyloglucan	Szymańska-Chargot et al. (2016)
1525	C=C stretching	Lignin	Stevanic and Salmen (2009)
		Chlorophyll	Koyama et al. (1986)
1615	C=C/C=N stretching	Chlorophyll	Koyama et al. (1986)
1650	C=C/C=O stretching	Chlorophyll	Koyama et al. (1986)

Cellulose is one of the main constituents of the cell wall surrounding the plasma membrane of plant cells. In the analyzed 700–1,800-cm^−1^ region, most cellulose-related bands overlap with or are masked by more intense signals from other cellular components. Consequently, the presence of cellulose was evaluated through secondary diagnostic bands, notably at approximately 1,090 cm^−1^ (skeletal C−O−C stretching of the β-(1→4)-glycosidic linkage), 1,155 cm^−1^ (C−O−C stretching of the β-(1→4)-glycosidic bridge), and 1,415 cm^−1^ (CH_2_ scissoring of the hydroxymethyl group). The disappearance or marked reduction of the 1,155- and 1,415-cm^−1^ bands in protoplast spectra indicates the removal of cellulose during enzymatic digestion.

In addition to cellulose, other cell wall components were identified by Raman spectroscopy, in particular xyloglucan, pectin, and lignin. Xyloglucan fingerprint bands were detected at 780 cm^−1^ (pyranose ring deformation coupled with C−O−C skeletal bending) and 1,480 cm^−1^ (CH_2_/CH bending), with the 1,480-cm^−1^ band showing a decrease in protoplasts compared with embryogenic calli. In the case of the strong band at 890 cm^−1^, the signal of xyloglucan is actually covered by the relatively more intense signal of pectin (C1−O−C4 stretching of the α-(1→4) glycosidic bond), which persists after cell wall removal.

The presence of pectin, or pectin-related materials, can be caused by the digestion process used to isolate the protoplast. The retention of pectin-related signals after digestion is further supported by the band at 1,455 cm^−1^ (CH_2_ methylene scissoring and CH methine deformation). Unlike other pectin-related peaks, the signal at 1,455 cm^−1^ is specifically associated with low-methylated pectin molecules, likely reflecting soluble fragments or residual wall components released during the enzymatic isolation process.

Clear differences were also observed in lignin-associated bands. Strong peaks at 1,272 cm^−1^ (aryl−*O* phenolic ether stretching coupled with C−O stretching in methoxy groups) and 1,477 cm^−1^ (asymmetric bending of methoxy –OCH_3_ groups) were clearly detectable in embryogenic calli, whereas their signals were markedly reduced in protoplasts. In the 1,600- to 1,700-cm^−1^ region, several overlapping bands were observed, corresponding to contributions from coniferyl aldehyde, coniferyl alcohol units, and other chromophores.

Vibrational contributions from starch were also identified. By comparing the spectra of **Figure 3** with reference spectra of starches (Puppulin et al., 2017), many similarities can be noted, particularly in the 1,000- to 1,500-cm^−1^ region, owing to the structural similarity between cellulose and starch as glucose polymers. Since plant cells synthesize and accumulate starch, the recorded spectra reflect overlapping contributions from both polymers.

### 3.4 Transient gene expression analysis

To monitor whether cell wall removal was effective and allowed for efficient protoplast transfection, we transfected protoplasts of both cultivars with PEG using a concentration of 10^5^ protoplasts/mL and a vector carrying the GFP marker gene (pEGB3Ω2-35S::GFP::Tnos). The GFP signal appeared in transfected protoplasts 24 h posttransfection in both cultivars (**Figures 4A–D**), confirming that the degree of cell wall removal monitored by RS was functionally sufficient for PEG-mediated delivery of exogenous material. Transfection with the empty vector as a negative control (**Figures 4E, F**) showed an absence of GFP signal.

**Figure 4:**
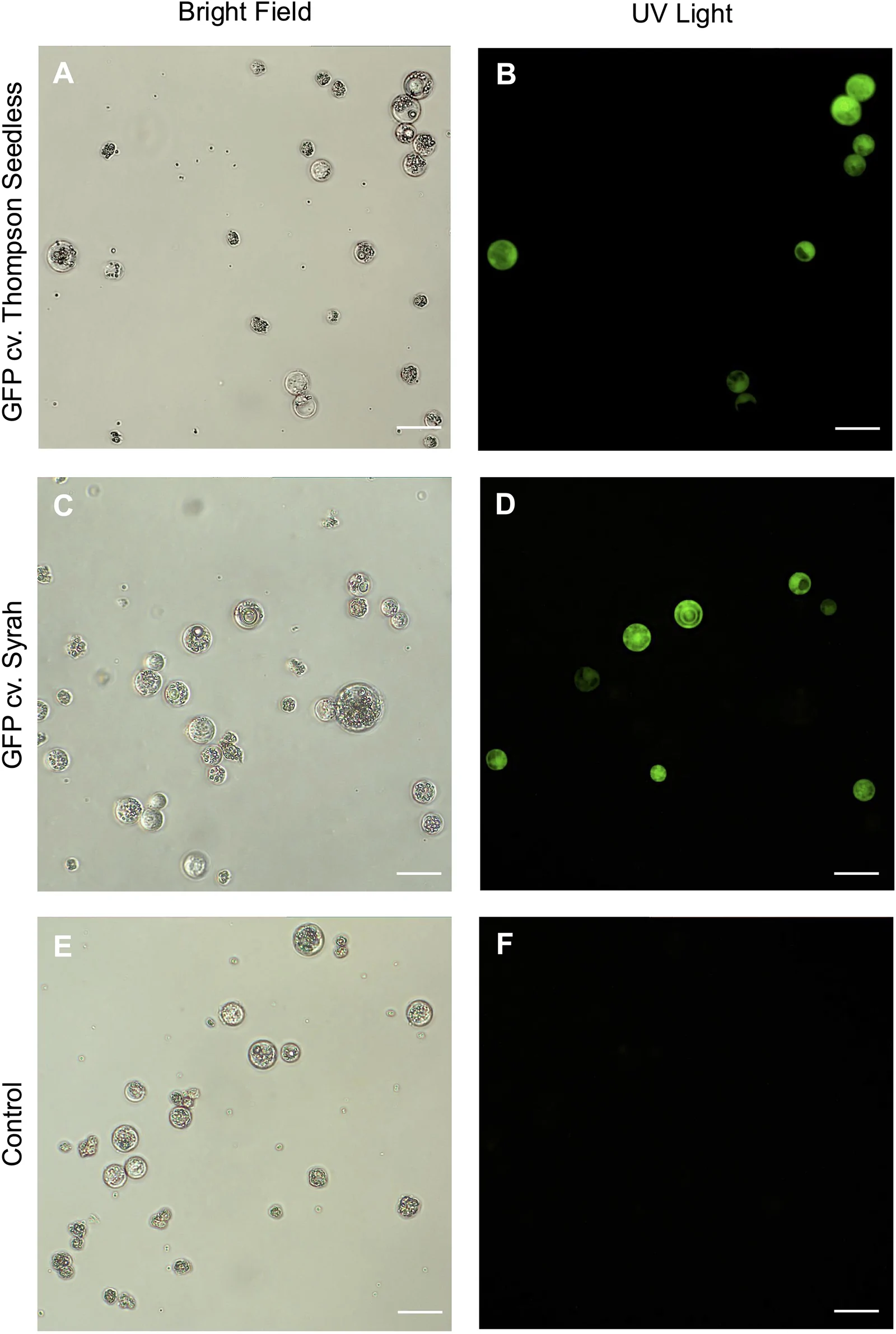
Transient expression of GFP in *Vitis vinifera* protoplasts. Protoplasts of cv. Thompson Seedless transfected with the pEGB3Ω2-35S::GFP::Tnos vector (GFP) after 24 h in bright field **(A)** and under UV light **(B)**. Protoplasts of cv. Syrah transfected with the pEGB3Ω2-35S::GFP::Tnos vector (GFP) after 24 h in bright field **(C)** and under UV light **(D)**. Protoplasts transfected with the empty vector (control) after 24 h in bright field **(E)** and under UV light **(F)**. Scale bar = 20 µm.

### 3.5 Protoplast cultivation and plant regeneration

To functionally validate the quality and developmental competence of the isolated protoplasts, we subsequently evaluated their ability to undergo somatic embryogenesis and regenerate whole plants. Isolated protoplasts (**Figure 5A**) were cultivated at three different concentrations (1 × 10^5^, 5 × 10^5^, and 10^6^ protoplasts/mL plating density) using the disc-culture method. Regeneration steps and somatic embryo development were monitored every week, and the timing was similar in both cultivars. The first cell division occurred 2 weeks after protoplast isolation (**Figure 5B**), microcolonies were formed after 4 weeks after protoplast isolation (**Figure 5C**), and the first embryos at the globular and heart stages appeared during the sixth and seventh weeks (**Figures 5D, E**). After around 3 months, mature cotyledonary embryos were regenerated (**Figure 5F**).

**Figure 5:**
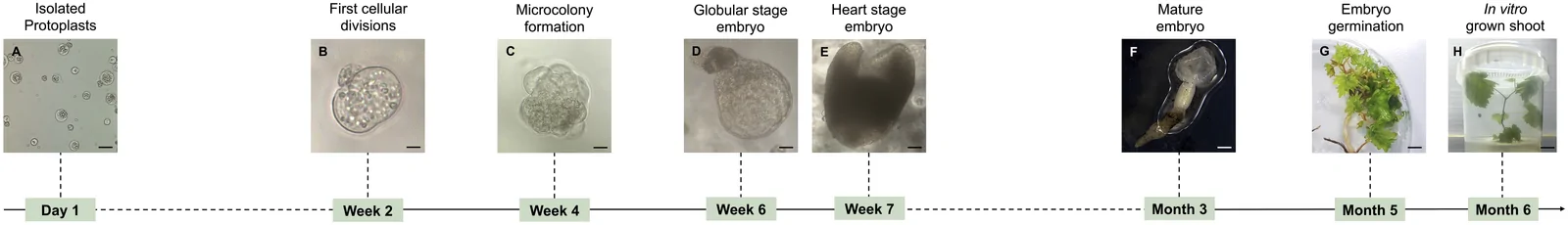
Protoplast-derived plant regeneration in *Vitis vinifera*. **(A)** Isolated protoplasts. Scale bar = 20 µm. **(B)** First cellular divisions appearing ~ 2 weeks after protoplast isolation. Scale bar = 30 µm. **(C)** Microcolony formation appearing ~ 4 weeks after protoplast isolation. Scale bar = 40 µm. **(D)** Globular stage of embryo development appearing ~ 6 weeks after protoplast isolation. Scale bar = 50 µm. **(E)** Heart stage of embryo development appearing ~ 7 weeks after protoplast isolation. Scale bar = 70 µm. **(F)** Cotyledonal stage mature embryo appearing ~ 3 months after protoplasts isolation. Scale bar = 0.5 mm. **(G)** Emerging shoot during embryo germination appearing ~ 5 months after protoplasts isolation. Scale bar = 5 mm. **(H)** *In vitro* plantlet appearing ~ 5 months after protoplasts isolation after exposure to light. Scale bar = 2 cm.

Regarding their regeneration, a concentration of 1 × 10^5^ protoplasts/mL was the most efficient for both cultivars, resulting in the highest recovery of mature cotyledonary embryos, followed by concentrations of 5 × 10^5^ and 1 × 10^6^ protoplast/mL (**Table 2**). The mean number of mature embryos in cv. Syrah was approximately twice that of cv. Thompson Seedless across all the concentrations used, indicating that Syrah was significantly more efficient than Thompson Seedless in mature somatic embryo development (**Table 2**).

**Table 2:** Effect of protoplast plating density on somatic embryo development, embryo germination, and whole-plant regeneration from protoplasts of cv. Thompson Seedless and cv. Syrah.

Regeneration steps	Protoplasts concentration (ppt/mL)	Total cultivated protoplasts	cv. Thompson seedless (mean ± SD)	cv. Syrah (mean ± SD)
No. of mature embryos	1 × 10^5^	1.2 × 10^6^	25 ± 4 a	57 ± 8^**^ a
	5 × 10^5^	6 × 10^6^	17 ± 3 b	33 ± 3^**^ b
	1 × 10^6^	12 × 10^6^	15 ± 3 b	30 ± 4^**^ b
No. of germinated embryos	1 × 10^5^	1.2 × 10^6^	12 ± 3 a	37 ± 4^**^ a
	5 × 10^5^	6 × 10^6^	8 ± 3 a	29 ± 2^***^ ab
	1 × 10^6^	12 × 10^6^	8 ± 1 a	23 ± 4^**^ b
No. of whole plants	1 × 10^5^	1.2 × 10^6^	4 ± 2 a	12 ± 4^*^ a
	5 × 10^5^	6 × 10^6^	3 ± 1 a	10 ± 3^*^ a
	1 × 10^6^	12 × 10^6^	4 ± 1 a	5 ± 1^ns^ a

The total number of cultivated protoplasts was calculated from three plates, with four 1-mL samples per plate (**Supplementary Figure 1**). For further details on shoot regeneration and whole-plant regeneration, see **Supplementary Table 3**. Data are expressed as mean ± SD (*n*=3). For each regeneration phase, different comparisons were performed between cultivars at the same protoplast concentration (Student’s t-test; *p<0.05; **p<0.01; ***p<0.001; ns, not significant) and among protoplast concentrations within the same cultivar (ANOVA test, α=0.05 followed by a Tukey’s HSD *post hoc* test; different lowercase letters within the same column (cultivar) and regeneration phase indicate statistically significant differences among protoplast concentrations. Values sharing the same letter are not significantly different).

Regenerated mature embryos were then transferred to germination medium. The germination capability of cv. Syrah somatic embryos was significantly higher than that of cv. Thompson Seedless across all concentrations (**Table 2**). For each plating density, around half of the mature embryos of cv. Thompson Seedless were well-germinated, while in cv. Syrah, more than two-thirds of the somatic embryos germinated properly (**Table 2**). No significant differences were found in cv. Thompson Seedless across all concentrations, whereas in cv. Syrah, the concentration of 1 × 10^5^ protoplasts/mL resulted in significantly higher germination than 1 × 10^6^ protoplasts/mL (**Table 2**).

Thereafter, germinated embryos were transferred to shoot-inducing medium and, to identify the best shoot-induction conditions, four types of media were evaluated by adding the synthetic plant hormone BAP to the basal media C2D and MG1 (**Supplementary Table 3**). For each protoplast concentration, the total number of germinated embryos was divided into four equal groups and transferred to each medium. For both cultivars at each concentration, except cv. Thompson Seedless at 1 × 10^6^ protoplast/mL, the highest number of regenerated shoots was obtained using MG1 + 10B medium (**Supplementary Table 3**).

To promote plant development, shoots were transferred from MG1/MG1 + 10B to RIM and from C2D/C2D + 4B to MSN (**Figure 5G**; **Supplementary Table 3**). All shoots in both media developed into whole plants with expanded leaves and roots (**Figure 5H**; **Table 2**; **Supplementary Table 3**). Regarding whole-plant regeneration from germinated embryos, the number of regenerated plants of cv. Syrah for 1 × 10^5^ and 5 × 10^5^ protoplasts/mL was significantly higher than that of cv. Thompson Seedless, whereas no significant differences were observed at 10^6^ protoplasts/mL (**Table 2**). No significant differences in plating density efficiency were observed among the three concentrations in both cultivars (**Table 2**).

Plants were finally transferred to the greenhouse and, after a period of acclimation, showed normal growth and morphology in both cultivars (**Supplementary Figure 3**).

Overall, these results showed that the most efficient protoplasts concentration for plant regeneration was 1 × 10^5^ protoplasts/mL, and an inverse relationship was observed between protoplast concentration and the number of regenerated plants.

## 4 Discussion

Grapevine, one of the world’s most economically important perennial crops, is increasingly threatened by climate change and emerging pathogens, making the development of improved cultivars a major priority and highlighting the potential of new genomic techniques, particularly DNA-free genome editing based on protoplast transfection, as demonstrated by recent successful applications in grapevine (Scintilla et al., 2022; Najafi et al., 2023; Gambino et al., 2024; Tricoli and Debernardi, 2024; Böttcher et al., 2025). However, the efficiency of these approaches is still limited, and several factors may contribute to reduced performance, including the quality of the initial protoplast preparation, genotype-dependent recalcitrance, and the intrinsic complexity of the whole-plant regeneration process (Nerva et al., 2023).

One of the critical steps in protoplast-based systems is the removal of the cell wall during enzymatic digestion, and residual cell wall components may negatively affect subsequent processes such as transformation efficiency and plant regeneration. Cell wall degradation is generally monitored using histological staining (e.g., Calcofluor White, Fluorescent Brightener 28) combined with microscopic observations, which allow for verifying the efficient removal of key cell wall components (Yang et al., 2008; Dai et al., 2010; Monthony and Jones, 2024; Stelate and Shik, 2026). A detailed analysis of the structure and composition of the plant cell wall during protoplast isolation is therefore essential to evaluate the extent of cell wall degradation. In the present study, RS is introduced as a rapid, label-free, and nondestructive approach to assess cell wall removal and ensure high-quality protoplast preparations prior to downstream applications (**Figure 6**).

**Figure 6:**
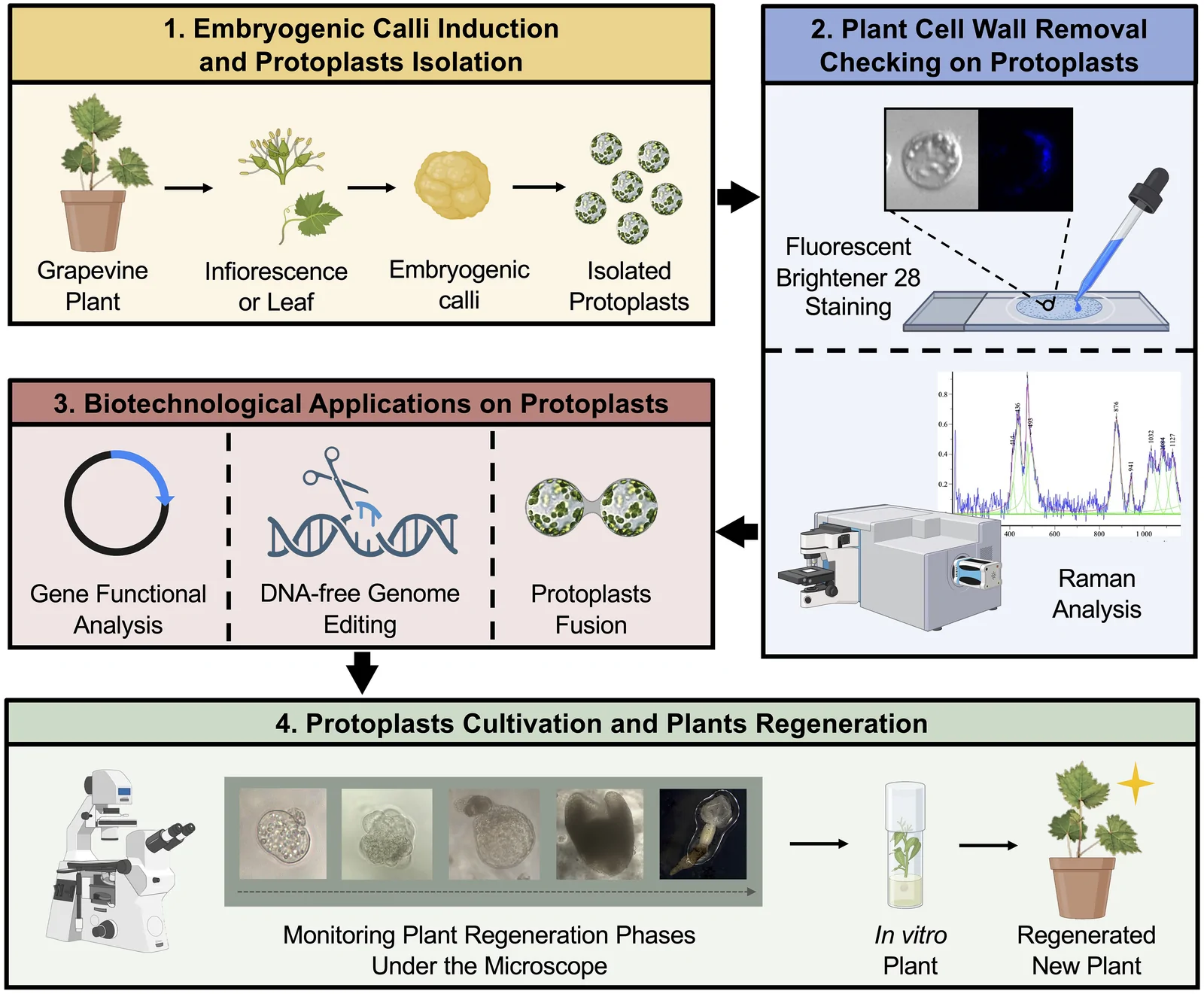
Schematic overview of the optimized grapevine protoplast-to-plant platform integrated with Raman spectroscopic quality control. The experimental workflow comprises four distinct phases: (1) Embryogenic calli induction and protoplast isolation from inflorescences or leaves; (2) cell wall removal validation by comparing conventional Fluorescent Brightener 28 staining with noninvasive Raman spectroscopy—as an upstream quality-control checkpoint to validate complete cell wall degradation; (3) downstream biotechnological applications, including functional genomics, DNA-free genome editing, and protoplast fusion; and (4) downstream biological validation through somatic embryogenesis (from cell division to the cotyledonary stage), followed by *in vitro* plantlet development and successful greenhouse acclimatization to confirm protoplast totipotency and developmental competence.

The protoplast–plant regeneration system is described for two important grapevine cultivars, Thompson Seedless and Syrah. Both cultivars have already been used to apply DNA-free genome editing by protoplast transfection with RNP and to regenerate transgene-free protoplast-derived plants (Najafi et al., 2023; Tricoli and Debernardi, 2024; Böttcher et al., 2025). In both cultivars, protoplasts were isolated from embryogenic calli, which are widely regarded as the most suitable tissue source of regenerable protoplasts in grapevine. Embryogenic cultures are generally induced from floral explants, particularly stamens and pistils. However, in seedless cultivars, including Thompson Seedless, embryogenic calli can also be obtained from young, still-unfolded leaves collected from *in vitro*-grown shoots (Dhekney et al., 2009). The establishment and maintenance of embryogenic cultures therefore represented critical steps in the success of the protoplast-to-plant system. Previous studies demonstrated that C1^P^ medium is suitable for long-term maintenance of grapevine embryogenic cultures, supporting sustained growth while preserving their regeneration competence (Iocco et al., 2001). Accordingly, in the present study, C1^P^ medium was used to propagate the embryogenic calli of both cultivars for approximately 1 year, while maintaining a high embryogenic potential.

Protoplasts were isolated from embryogenic calli after 7 days of subculturing in C1^P^ medium. Regarding cv Syrah, protoplast isolation was previously described in another study (Böttcher et al., 2025). In comparison with this study, the final yield described here is higher (6.9 × 10^7^ cell/g embryogenic calli rather than 1 × 10^6^ cell/2 g embryogenic calli). This result could be attributed to the shorter incubation time of the embryogenic calli in the digestion solution and the absence of a sucrose gradient purification step compared with the protocol described by Böttcher et al. (2025). Regarding Thompson Seedless, the yield of isolated protoplasts is comparable to that reported in Tricoli and Debernardi (2024).

Raman spectroscopy analysis applied to embryogenic calli and isolated protoplasts of both cultivars revealed consistent structural differences resulting from the enzymatic digestion treatments. In particular, the disappearance or marked reduction of bands associated with cellulose at approximately 1,155 and 1,415 cm^−1^, together with the decrease in the xyloglucan-associated band at approximately 1,480 cm^−1^ and the reduction of the lignin-associated band around 1,272 cm^−1^, clearly indicate the degradation of structural cell wall components. The reduction or absence of these characteristic signals in protoplast spectra of both cultivars, together with Fluorescent Brightener 28 staining results and successful PEG-mediated GFP transfection, demonstrates the effectiveness of the enzymatic digestion process and validates Raman spectroscopy as a nondestructive tool to monitor cell wall removal. Therefore, the bands at 1,155, 1,272, 1,415, and 1,480 cm^−1^ may represent candidate Raman spectral markers for monitoring cell wall removal during grapevine protoplast isolation.

While conventional dye staining remains the standard for routine protoplast verification, RS offers an objective, label-free quality-control method that overcomes visual artifacts and operator subjectivity. By providing a distinct molecular signature of cell wall removal, RS is particularly valuable when optimizing and validating protoplast isolation protocols in new plant species, genotypes, or tissue sources before downstream applications.

In future studies, the noninvasive nature of RS also offers a promising tool to monitor cell wall resynthesis kinetics during early protoplast culture, a critical determinant of cell division, microcolony formation, and long-term regeneration competence.

To evaluate downstream biological competence following quality-controlled isolation, protoplasts of cv. Thompsons Seedless and cv. Syrah were cultivated at three different concentrations. Protoplast plating density significantly influenced somatic embryo development: among the tested conditions, the lowest density (1 × 10^5^ protoplasts/mL) resulted in the highest number of mature and well-germinated somatic embryos in both cultivars (**Table 2**). Higher densities likely led to increased competition for nutrients, resulting in reduced embryogenesis efficiency. Regarding whole-plant regeneration, all successfully induced shoots converted into complete, rooted plantlets across all conditions (**Supplementary Table 3****),** and no significant differences were observed among the three tested conditions for both cultivars (**Table 2**). Overall, marked differences between the two cultivars were observed, with cv. Syrah showing significantly higher somatic embryo development, germination rate, and plant regeneration capacity compared with cv. Thompson Seedless across all evaluated concentrations (**Table 2**). Genotype-dependent regeneration has already been reported in the literature (Bertini et al., 2019; Dal Santo et al., 2022; Böttcher et al., 2025) and is likely driven by endogenous characteristics of the variety itself (e.g., epigenetic traits) as well as exogenous factors (e.g., varying responses to culture media, *in vitro* growth conditions, and other environmental factors).

Overall, while protoplast-to-plant regeneration protocols have been previously described in grapevine (Scintilla et al., 2022; Najafi et al., 2023; Tricoli and Debernardi, 2024; Böttcher et al., 2025), the primary methodological advance of the present study resides in establishing Raman spectroscopy as a non-invasive, label-free quality checkcontrol method for assessing the effectiveness of cell wall removal during protoplast isolation. In this context, successful somatic embryogenesis and whole-plant regeneration provide crucial biological validation, demonstrating that the RS-monitored protoplasts retained developmental competence and totipotency. Rather than replacing routine procedures, RS provides an objective assessment to support protocol consistency prior to key applications, such as transformation and genome editing. Using RS as a supportive quality-control step to validate protoplast pipelines enhances workflow reliability and reproducibility, aligning with evolving regulatory frameworks such as Regulation (EU) 2026/1388 for plants with NGT-1 status.

## 5 Conclusion

In conclusion, this study establishes RS as a rapid, label-free, and nondestructive quality-control tool during protoplast isolation in grapevine (*Vitis vinifera*). By identifying specific spectral markers corresponding to cellulose, xyloglucan, and lignin degradation, RS provides an objective assessment of cell wall removal, overcoming the visual artifacts and operator subjectivity inherent in traditional dye staining. Rather than replacing routine procedures, RS serves as an upstream standardization checkpoint, while downstream whole-plant regeneration in cv. Thompson Seedless and cv. Syrah confirms that the monitored protoplasts retain full cell viability, totipotency, and developmental competence.

While this study demonstrates the effectiveness of RS in grapevine, extending this spectral framework to additional genotypes and tissue sources represents the next step in validating its versatility. In future studies, applying RS to track cell wall resynthesis kinetics during early culture offers a promising strategy to better understand and optimize protoplast division and microcolony formation. Overall, integrating RS as an objective quality check enhances workflow reliability and reproducibility, supporting the optimization of protoplast platforms for generating transgene-free edited plants eligible for NGT-1 status.

## Data Availability

The raw data supporting the conclusions of this article will be made available by the authors, without undue reservation.
