# Supplementary material for: A novel application of Raman spectroscopy to monitor cell wall removal during protoplasting in grapevine
Source: Front Plant Sci. 2026 Sep 18;17:1940456. doi: 10.3389/fpls.2026.1940456 (PMC13644691; doi:10.3389/fpls.2026.1940456)
Supplement: Supplementary file 1 [file DataSheet1.pdf]

Supplementary Material

Supplementary Tables

Table S1. List of culture media with the respective composition. All components are provided by Duchefa Biochemie BV, except for phytagel (Merk).

Name	Abbreviation	Composition	Reference
**Culture medium for induction of embryogenic calli from *in-vitro* leaves**	NB2	NN macronutrients, NN micronutrients, MS vitamins, 1.0 µM 6-BAP, 5.0 µM 2,4-D, 0.1 g/L myo-inositol, 20 g/L sucrose, 7 g/L Agar-TC, pH = 6.0	(Dhekney et al., 2009)
**Culture medium for induction of embryogenic calli from stamens**	PIV	NN macronutrients, MS micronutrients, FeEDTA, B5 vitamins, 8.9 µM 6-BAP, 4.5 µM 2,4-D, 60 g/L sucrose, 3 g/L phytagel, pH = 5.7	(Franks et al., 1998)
**culture medium for long-term maintenance of embryogenic calli**	$C1^P$	MS macronutrients, MS micronutrients, FeEDTA, vitamins mix C1, amino acid mix, 1 µM 6-BAP, 5 µM 2,4-D, 1 g/L casein hydrolysate, 30 g/L sucrose, 5 g/L phytagel, pH = 5.8	(Iocco et al., 2001)
**Solid culture medium for protoplasts cultivation**	P-SM	NN macronutrients, NN micronutrients, NN vitamins, 10µM NAA, 2 µM 6-BAP, 30 g/L sucrose, 0.3M glucose, 2 g/L gelrite, pH = 5.7	(Zhu et al., 1997)
**Liquid culture medium for protoplasts cultivation**	P-LM	NN macronutrients, NN micronutrients, NN vitamins, 10µM NAA, 2 µM 6-BAP, 30 g/L sucrose, 0.3 M glucose, 3 g/L activated charcoal, pH = 5.7	(Zhu et al., 1997)
**Culture medium for somatic embryos full germination**	SE-M	NN macronutrients, NN micronutrients, NN vitamins, 30 g/L sucrose, 2 g/L gelrite, pH = 5.8	(Zhu et al., 1997)
**Culture media for somatic embryos shooting**	C2D	C2D macronutrients, C2D micronutrients, C2D vitamins, 30 g/L sucrose, 7 g/L agar TC, pH = 5.8	(Li et al., 2014)
	C2D+4B	C2D macronutrients, C2D micronutrients, C2D vitamins, 5µM 6-BAP, 30 g/L sucrose, 7 g/L agar TC, pH = 5.8	(Li et al., 2014)
	MG1	NN macronutrients, MS micronutrients, Fe-EDTA, B5 vitamins, 30 g/L sucrose, 7 g/L agar-TC, 2.5 g/L activated charcoal, pH = 5.8	(Torregrosa et al., 2015)

	MG1+10B	NN macronutrients, MS micronutrients, Fe-EDTA, B5 vitamins, 5µM 6-BAP, 30 g/L sucrose, 7 g/L agar-TC, 2.5 g/L activated charcoal, pH = 5.8	(Torregrosa et al., 2015)
**Culture media for full plant development and rooting**	MSN	MS macronutrients, MS micronutrients, MS vitamins, 0.5 µM NAA, 30 g/L sucrose, 7 g/L agar-TC, pH = 5.8	(Li et al., 2014)
	RIM	MS macronutrients, MS micronutrients, Fe-EDTA, Vitamins T, 0.5 µM NAA, 30 g/L sucrose, 7 g/L agar-TC, pH = 6.0	(Torregrosa et al., 2015)

Table S2. List of solution used for protoplast isolation, purification and transfection with the respective composition. All components are provided by Duchefa Biochemie BV.

**Name**	**Composition**	**Reference**
**Digestion solution**	2% (w/V) Cellulase Onozuka R-10, 1% (w/V) Macerozyme R10, 0.05% (w/V) Pectolyase Y23, 0.5 M Mannitol, 10 mM $CaCl_2x2H_2O$, 5 mM 2-(N-morpholino) ethanesulfonic acid [pH = 5.7]	(Zhu et al., 1997)
**Wash solution**	0.5 M Mannitol, 10 mM $CaCl_2x2H_2O$	(Zhu et al., 1997)
**MMG**	0.4 M Mannitol, 15 mM $MgCl_2x6H_2O$, 4 mM 2-(N-morpholino) ethanesulfonic acid [pH = 5.7]	(Woo et al., 2015)
**PEG**	0.2 M Mannitol, 100 mM $CaCl_2x2H_2O$, 40% w/V PEG 4000	(Woo et al., 2015)
**W5**	125 mM $CaCl_2x2H_2O$, 2 mM 2-(N-morpholino) ethanesulfonic acid [pH = 5.7], 154 mM NaCl, 5 mM KCl	(Woo et al., 2015)
**W1**	0.5 M Mannitol, 20 mM KCl, 4 mM 2-(N-morpholino) ethanesulfonic acid [pH = 5.7]	(Woo et al., 2015)

Table S3. Effect of protoplast plating density and culture medium on shoot and whole-plant regeneration from protoplasts of cv. Thompson Seedless and cv. Syrah. The total number of cultivated protoplasts was calculated based on three plates, with four 1-mL drops per plate (see **Fig. S1**). Data are reported as the total number of regenerated shoots or whole plants obtained from the three independent biological replicates. The corresponding mean ± SD values are reported in **Table 2**.

	**Protoplasts concentration (ppt/mL)**	**N° germinated embryos**	**N° regenerated shoots**				**N° whole plants**	
			**MG1**	**MG1 +10B**	**C2D**	**C2D +4B**	**RIM** (from MG1)	**MSN** (from C2D)
cv. Thompson Seedless	**$1x10^5$**	**36**	3/9	4/9	2/9	2/9	7/7	4/4
	**$5x10^5$**	**24**	2/6	3/6	2/6	1/6	5/5	3/3
	**$1x10^6$**	**24**	2/6	4/6	6/6	0/6	6/6	6/6
			7 tot	*11 tot*	*10 tot*	*3 tot*	*18 tot*	*13 tot*
cv. Syrah	**$1x10^5$**	**112**	8/28	16/28	7/28	5/28	24/24	12/12
	**$5x10^5$**	**88**	5/22	13/22	4/22	7/22	18/18	11/11
	**$1x10^6$**	**68**	0/17	10/17	4/17	2/7	10/10	6/6
			13 tot	*39 tot*	*15 tot*	*14 tot*	*52 tot*	*29 tot*

Supplementary Figures

Figure S1. Experimental design for protoplasts (ppt) cultivation at different plating densities using the disc-culture method. Schematic representation of the three tested protoplasts concentrations (1×10^5, 5×10^5 and 1×10^6 protoplasts/mL) cultured in solid P-SM medium and supplemented with liquid P-LM medium as a reservoir. For each concentration, four 1 mL drops were prepared per replicate and three replicates were performed (12 drops in total), corresponding to a total number of 1.2×10^6, 6×10^6 and 12×10^6 cultured protoplasts, respectively.

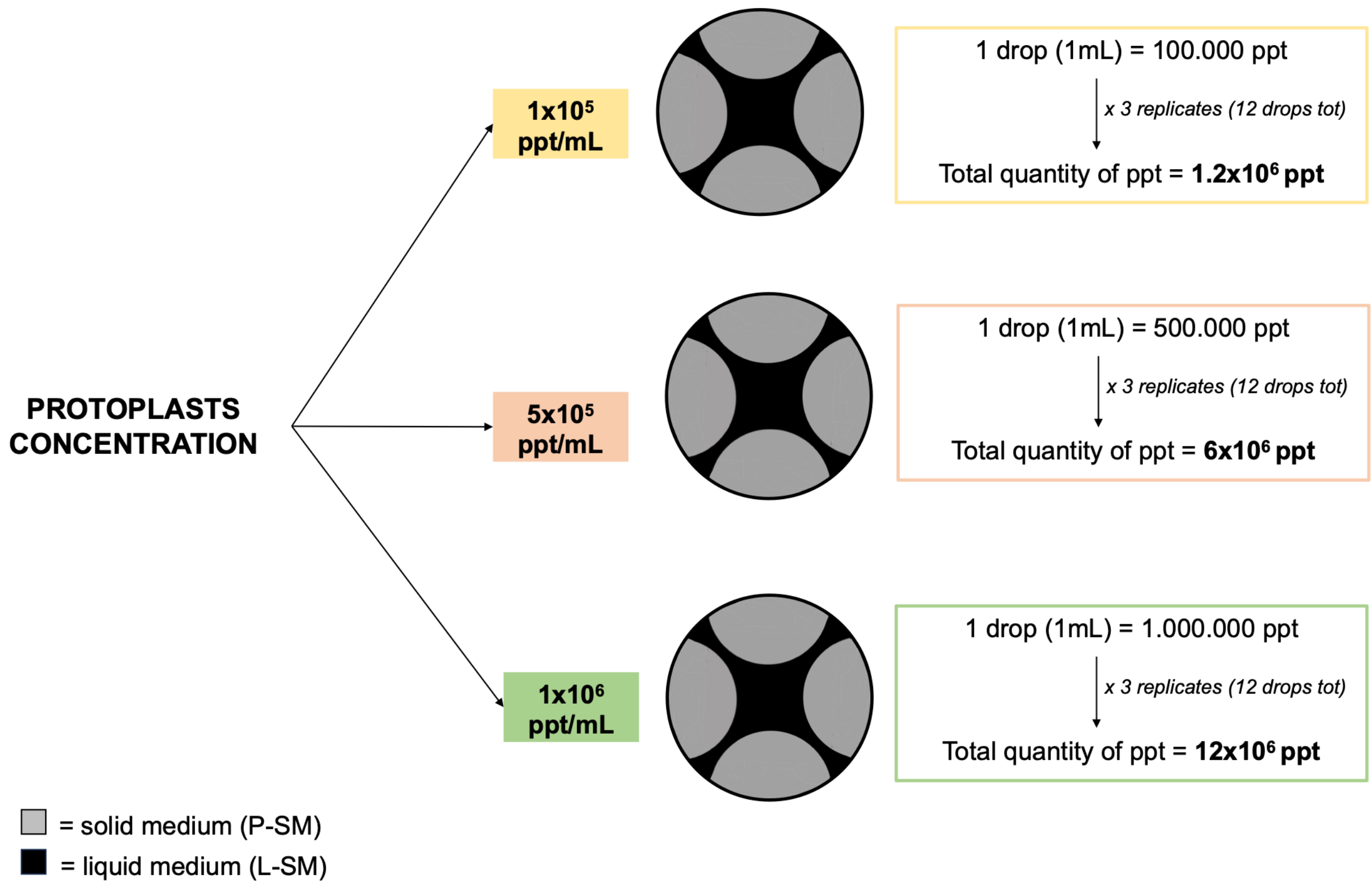

Figure S2. Evaluation of viability and cell wall digestion efficiency on isolated protoplasts. **(A)** Protoplasts of cv. Thompson Seedless and Syrah observed in bright field (left) and after FDA staining (right) for analysis of viability efficiency. **(B)** Protoplasts of cv. Thompson Seedless and Syrah observed in bright field (left) and after Fluorescent Brightenere 28 staining (right) for analysis of cell wall digestion efficiency. Scale bar = 20 µm.

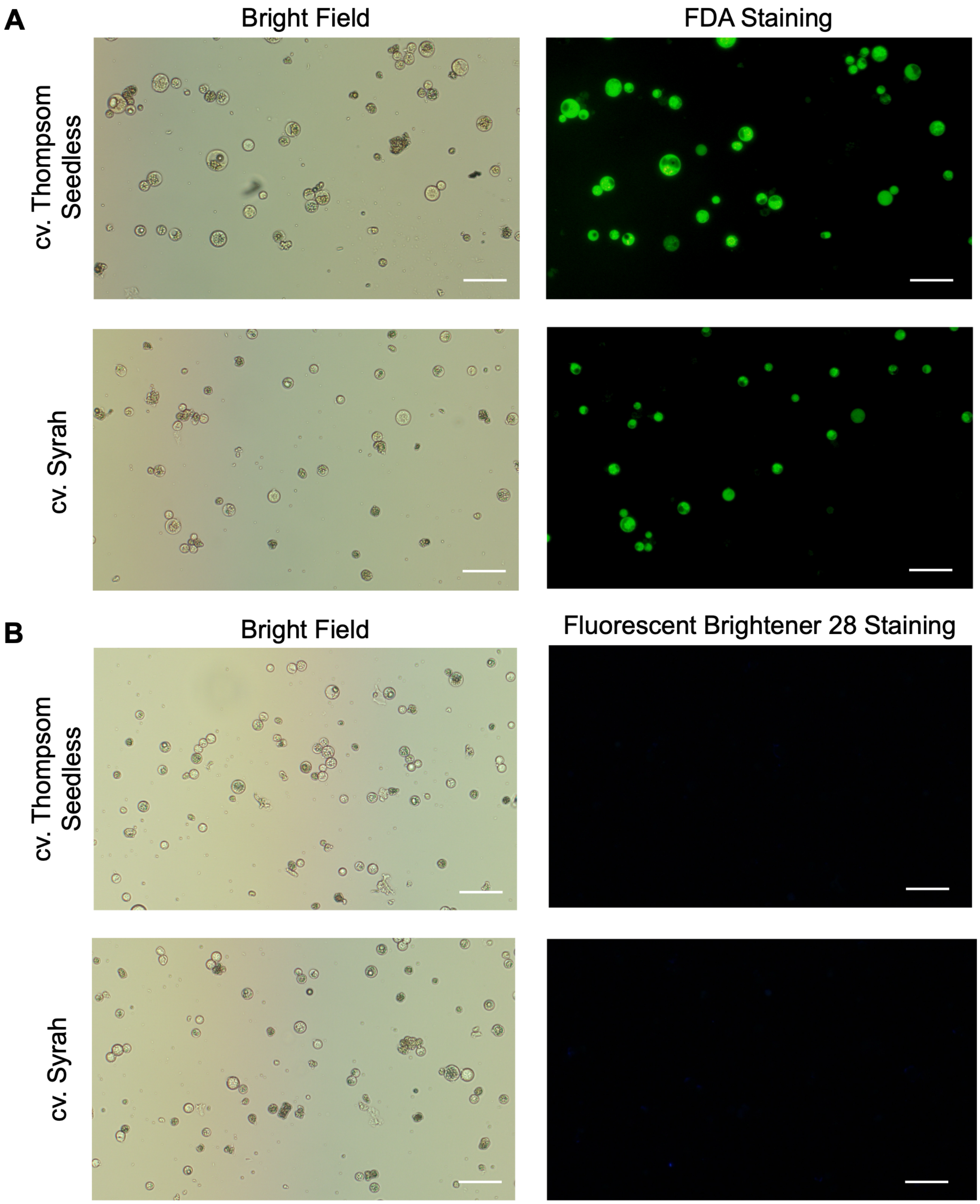

Figure S3. Regenerated plants after acclimation under greenhouse conditions. **(A)** Whole plant of cv. Thompson Seedless and **(B)** whole plant of cv. Syrah.

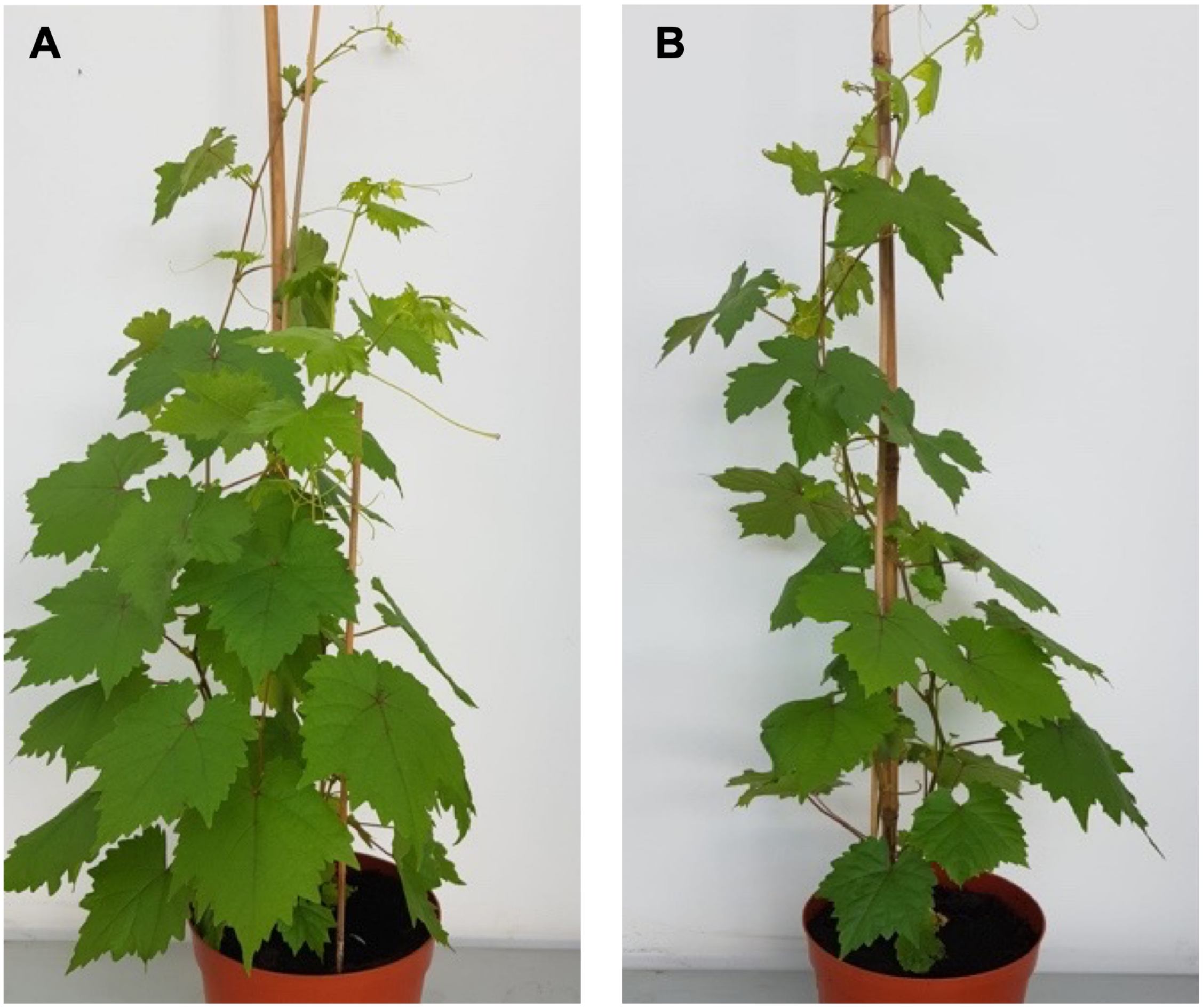

cv. Thompson Seedless

cv. Syrah

References

Dhekney, S.A., Li, Z.T., Compton, M.E., and Gray, D.J. (2009). Optimizing Initiation and Maintenance of Vitis Embryogenic Cultures. *HortScience* 44(5)**,** 1400-1406. doi: 10.21273/HORTSCI.44.5.1400.

Franks, T., Gang He, D., and Thomas, M. (1998). Regeneration of transgenic shape Vitis vinifera L. Sultana plants: genotypic and phenotypic analysis. *Molecular Breeding* 4(4)**,** 321-333. doi: 10.1023/A:1009673619456.

Iocco, P., Franks, T., and Thomas, M.R. (2001). Genetic Transformation of Major Wine Grape Cultivars of Vitis Vinifera L. *Transgenic Research* 10(2)**,** 105-112. doi: 10.1023/A:1008989610340.

Li, Z.T., Kim, K.-H., Dhekney, S.A., Jasinski, J.R., Creech, M.R., and Gray, D.J. (2014). An optimized procedure for plant recovery from somatic embryos significantly facilitates the genetic improvement of Vitis. *Horticulture Research* 1. doi: 10.1038/hortres.2014.27.

Torregrosa, L., Vialet, S., Adivèze, A., Iocco-Corena, P., and Thomas, M.R. (2015). Grapevine (Vitis vinifera L.). *Methods Mol Biol* 1224**,** 177-194. doi: 10.1007/978-1-4939-1658-0_15.

Woo, J.W., Kim, J., Kwon, S.I., Corvalán, C., Cho, S.W., Kim, H., et al. (2015). DNA-free genome editing in plants with preassembled CRISPR-Cas9 ribonucleoproteins. *Nature Biotechnology* 33(11)**,** 1162-1164. doi: 10.1038/nbt.3389.

Zhu, Y.-M., Hoshino, Y., Nakano, M., Takahashi, E., and Mii, M. (1997). Highly efficient system of plant regeneration from protoplasts of grapevine (Vitis vinifera L.) through somatic embryogenesis by using embryogenic callus culture and activated charcoal. *Plant Science* 123(1)**,** 151-157. doi: https://doi.org/10.1016/S0168-9452(96)04557-8.
